# Development and standardisation of ‘time-in-range’ measurement for clinical endpoints in retinal diseases

**DOI:** 10.1038/s41433-025-04112-6

**Published:** 2025-12-16

**Authors:** Luisa Frizziero, Jane Barratt, Chui Ming Gemmy Cheung, Andrii Korol, Edoardo Midena, James Talks, Miltiadis Tsilimbaris, Ian Pearce, Igor Kozak

**Affiliations:** 1 Department of Neuroscience, University of Padua, Padova, Italy; 2 Department of Ophthalmology, Padova University Hospital, Padova, Italy; 3 International Federation on Ageing, Toronto, ON Canada; 4 Singapore Eye Research Institute, Singapore National Eye Centre, Singapore, Singapore; 5 Duke-NUS Medical School, National University of Singapore, Singapore, Singapore; 6 The Filatov Institute of Eye Diseases and Tissue Therapy of the National Academy of Medical Sciences of Ukraine, Odesa, Ukraine; 7 IRCCS–Fondazione Bietti, Rome, Italy; 8 Newcastle upon Tyne Hospitals NHS Foundation Trust, Newcastle upon Tyne, UK; 9 Laboratory of Optics and Vision, University of Crete Medical School, Heraklion, Crete Greece; 10 Royal Liverpool University Hospital, Liverpool, UK; 11 Moorfields Eye Hospital Centre, Abu Dhabi, United Arab Emirates; 12 Department of Ophthalmology and Vision Science, University of Arizona, Tucson, Arizona USA

**Keywords:** Macular degeneration, Retinal diseases

## Abstract

Clear and updated endpoints are required to measure outcomes of a disease course and/or a therapeutic intervention. The aim of this review is to identify a reliable ‘time-in-range’ endpoint of clinical outcomes in ocular conditions, with a particular focus on exudative diseases involving the posterior pole of the eye, and to explore possible applications of this endpoint. A PubMed search was carried out pertaining to: ‘time-in-range’, ‘clinical-outcome’, ‘clinical-endpoint’, ‘clinical trial’, ‘metrics’, ‘retina’, ‘retinopathy’, ‘macular-oedema’, ‘maculopathy’, ‘ophthalmology’, ‘visual-function’, ‘visual acuity end-point’ and ‘OCT’. The results showed that both functional and morphological endpoints have been used in the evaluation of retinal diseases. At present, the most widely accepted and clinically meaningful marker of ocular disease is ‘mean change’ in best corrected visual acuity (BCVA). While comparisons to baseline at various timepoints are commonly recommended to evaluate statistical and clinically relevant differences, few metrics capture the disease course continuously over time. In other medical fields, ‘time-in-range’ has been introduced to provide more complete information on the fluctuations characterising the course of a disease. The application of ‘time-in-range’ on BCVA in exudative diseases involving the posterior pole seems feasible, reliable and applicable in clinical practice. BCVA ‘time-in-range’ offers a useful and practical endpoint in retinal diseases, evaluating both visual function at the end of an observation/treatment and fluctuations in disease over time. It may also be applied to other clinical and morphological endpoints in ocular diseases, including macular thickness. This review presents a hypothesis-generating framework proposing ‘time-in-range’ as a supplementary metric, pending prospective validation.

## Introduction

Significant technological progress has been made in the field of ophthalmology over the last 30 years, particularly in relation to retinal diseases [1]. Newly available technologies have allowed clinicians to better understand the mechanisms underlying retinal pathologies, such as diabetic retinopathy (DR) and diabetic macular oedema (DMO), age-related macular degeneration (AMD), inherited retinal diseases and glaucoma. As a result, several new therapeutic targets have been identified and the number of clinical trials has increased [1].

Measurable endpoints are crucial, both in clinical trials and clinical practice, to enable effective assessment of a disease course and/or a therapeutic intervention [2]. In ophthalmology, a range of clinical endpoints, both functional and morphological, have been used in late-phase trials with distinct strengths and weaknesses [3]. Most of the current clinical endpoints describe the final state of a longitudinal course, disregarding the relevance of possible fluctuations [3]. Recently, the new concept of ‘time-in-range’ (TIR) has been applied in diabetology and introduced in ophthalmology [4].

The aim of this review is to identify a possible reliable TIR endpoint of clinical outcomes in ophthalmology, with a particular focus on exudative diseases involving the posterior pole of the eye. We also propose possible future applications of this new endpoint.

## Methods

This article is based on a review of the literature and a consensus among retinal experts from the Vision Academy. The Vision Academy is a group of over 80 international experts who, through their collective expertise, provide consensus guidance for managing clinically challenging situations, especially in areas of controversy or with insufficient conclusive evidence (www.visionacademy.org). The Vision Academy is sponsored by Bayer.

The online PubMed database was searched using the following search terms (used both individually and in combination for advanced research): ‘time-in-range’, ‘clinical-outcome’, ‘clinical-endpoint’, ‘clinical trial’, ‘metrics’, ‘retina’, ‘retinopathy’, ‘macular-oedema’, ‘maculopathy’, ‘ophthalmology’, ‘visual-function’, ‘visual acuity end-point’ and ‘OCT’. Additional articles were identified by reviewing the references cited in examined publications. More than 200 publications published between August 2017 and October 2024 were reviewed to identify key studies showing important features related to the aim of the paper. Recommendations based on the literature search results and the authors’ own clinical experience were developed and subsequently reviewed, commented on and endorsed by a majority of the Vision Academy prior to finalisation. For each proposed recommendation, respondents were asked to rate their agreement using a five-point categorical scale: ‘strongly agree’, ‘agree’, ‘neither agree nor disagree’, ‘disagree’ and ‘strongly disagree’. Responses from more than 50% of the Academy’s membership were required for the survey to be valid. To assess any influence of the healthcare system on the survey responses, respondents were also asked for the reimbursement status of treatment in their country of practice (reimbursed, out-of-pocket or a combination of the two). Biases were assessed using χ^2^. Endorsement was established if 50% or more of respondents indicated that they agreed or strongly agreed with a recommendation; consensus was considered ‘strong’ if more than 75% of respondents agreed or strongly agreed. The list of Vision Academy members and mentees who contributed to the recommendations is provided at the end of this article.

## Endpoints: clinical, surrogate and biomarker

Endpoints are specific measures of outcomes occurring because of an intervention or the absence of an intervention [2]. In ophthalmology, endpoints are evaluated using functional metrics. Clinical endpoints relate to outcomes that capture how a person feels, functions or survives, and therefore have direct importance to patients and reflect their wellbeing and quality of life [1, 2]. These endpoints may be measured objectively or subjectively, and reported by clinicians, patients or observers, or assessed through performance measures [2]. Non-clinical endpoints do not relate directly to how a person feels, functions or survives, but are indicators of a biological or pathogenic process and are objectively measured and used for diagnostic, prognostic and/or monitoring purposes. These include, for example, imaging results, which may provide morphological metrics [2]. Surrogate endpoints do not directly measure how a person feels, functions or survives, but are considered reliable substitutes of clinical endpoints since they are closely associated with them and directly measure causal intermediaries of the effect of an intervention on a clinically meaningful outcome [2]. Therefore, they may include morphological biomarkers directly influencing visual function. However, the correlation between the marker and the visual benefit/loss should be confirmed, usually by longitudinal studies [1]. Endpoint characteristics should include the ability to capture the outcome of interest accurately (measure what is intended), precisely (with minimal error or uncertainty), and consistently with repeated measurements [2].

## The ‘time-in-range’ concept

One of the main limitations of most endpoints is that they may not consistently detect the effect of an intervention across all stages of disease [2]. It may be difficult for some endpoints, particularly surrogate ones, to determine what degree or duration of effect corresponds with a clinically meaningful effect. For example, even if lowering blood pressure is causally associated with a reduction in the risk of cardiovascular death, it may not be easy to establish the exact magnitude and duration of blood pressure reduction that translates into a quantifiable reduction in mortality risk [2]. An area-under-the-curve analysis may provide quantitative information over a period of time and, specifically, a measure of the changes of quantitative data over time [5]. However, the obtained result is an averaged measure over a period of time, without giving an idea of the fluctuations of that measure over that period or of the time spent within certain values [4].

The concept that the proportion of time spent within a certain range may be a realistic and reliable clinical marker, offering a more complete reflection of a disease than clinical measurements at single, limited timepoints, is currently well accepted in diabetes [6]. TIR derives from the introduction of continuous glucose monitoring, which captures the glucose profile over a number of days. It has allowed the introduction of new metrics beyond the sole glycated haemoglobin A1c (HbA1c), which provides information on mean glycaemic control over a period of time but not on its fluctuations during that period [7]. In fact, HbA1c failed, for example, to distinguish between conventional and intensive insulin treatment groups, which are characterised by a different risk of DR [7, 8]. In this context, TIR has been defined as the percentage of time an individual spends within the target glucose range, giving an idea of the frequency and duration of hypoglycaemia or hyperglycaemia over time. TIR has been related to microvascular complications of diabetes and all-cause mortality, and thus proposed as a clinical endpoint in clinical trials [7, 9, 10]. In particular, it is significantly associated with the prevalence of all stages of DR even after adjusting for HbA1c [7]. Therefore, TIR has been recommended as an integral part of the day-to-day management of diabetes mellitus [11].

The TIR concept is also already established in the management of patients receiving anticoagulants, where it is one of the key points in the American College of Chest Physicians (CHEST) guidelines [12]. Moreover, it is a concept that may be extended to other medical fields (e.g., the time of stability/a decrease in CD4+ T-cell count, which is associated with human immunodeficiency virus progression) [13].

Besides the clinical meaning of TIR, its application has also been shown to improve disease control due to a better understanding of disease behaviour in different patients, thus improving patient compliance to treatment [4, 7, 14].

## State-of-the-art clinical endpoints in ophthalmology

The clinical endpoints currently accepted in ophthalmology primarily include functional metrics [1]. Several new morphological metrics have been proposed, particularly since the advent of spectral-domain optical coherence tomography (OCT) and OCT angiography (OCT-A), showing a high correlation with visual prognosis and disease progression, particularly in DMO [15]. Some metrics have the advantage of easy repeatability, which is one of the key points to establishing robust, meaningful and standard clinical endpoints for interventional clinical trials [16]. Functional and morphological endpoints, as well as their indications, advantages and disadvantages, are shown in Tables 1 and 2, respectively.

**Table 1:** Visual function endpoints.

Endpoint	Test	Indications	Advantages	Disadvantages
BCVA	BCVA score Mean change in BCVA [21] Percentage of VA gain (>0, >5, >10, >15 letters) Percentage with VA >20/40 [50]	All ocular diseases	Widespread; inexpensive, rapid and intuitive to perform and understand; possibly self-administered; correlates with quality of life	Limited value in early disease and tracking small changes; does not consider BCVA changes during the study period
LLVA	LLVA score [27] Mean change in LLVA [20]	AMD, DMO, DR, CSCR, IRD	Simple, inexpensive and rapid; possibly self-administered	Selected information (foveal function); related to the timepoint assessed
Contrast sensitivity	Contrast sensitivity level (e.g., number of triplets on Pelli-Robson charts) Change in contrast sensitivity [20]	AMD, DMO, DR, refractive surgery, CSCR, IRD	Rapid and correlates with quality of life	Difficult to set up accurately; limited sensitivity; not well standardised and widespread; related to the timepoint assessed
Perimetry	Mean sensitivity and mean deviation [1]	Glaucoma, neurological conditions, retinal diseases	Comprehensive measure of visual function	Need for adequate instruments and time; influenced by the conditions of execution and the patient’s learning curve
Microperimetry	Scotopic/mesopic sensitivity [20]	AMD, DMO, vitreoretinal disorders, retinotoxicity disorders, macular dystrophies, IRD	Better understanding of morphology and function; particularly used to determine fixation in advanced AMD	Not widespread; long testing duration; related to the timepoint assessed; learning curve
Dark adaptometry	Change in visual performance [20, 21]	AMD, DMO, DR To differentiate AMD from variants of genetic disease Early diagnosis of AMD progression if short-duration testing strategies prove effective	Assesses photoreceptor dynamic response	Need for adequate instruments and time; lack of standardisation; related to the timepoint assessed
Reading speed	Mean change in reading speed [20]	AMD, DMO, vitreoretinal disorders, refractive surgery	Strongly linked to vision-related quality of life	Lack of standardisation and agreement on methodology; dependent on patient literacy; related to the timepoint assessed

*AMD* age-related macular degeneration, *BCVA* best corrected visual acuity, *CSCR* central serous chorioretinopathy, *DMO* diabetic macular oedema, *DR* diabetic retinopathy, *IRD* inherited retinal diseases, *LLVA* low-luminance visual acuity, *VA* visual acuity.

**Table 2:** Morphological endpoints.

Endpoint	Test	Indications	Advantages	Disadvantages
Geographic atrophy area	Geographic atrophy area Geographic atrophy area enlargement [1]	AMD	Accurate and rapid	Influenced by image quality; related to the timepoint assessed; for atrophic diseases only
Ellipsoid zone and/or external limiting membrane defects	Percentage of defect Rate of change in defect as measured by OCT [20]	Macular diseases	Well related to visual function	Influenced by image quality; related to the timepoint assessed; provides information on photoreceptor status only
Central subfield thickness	Mean thickness Change in thickness on OCT [1]	Macular diseases	Rapid, standardised and widespread	Weakly correlated with VA; limited area of evaluation (fovea and perifovea); related to the timepoint assessed
Macular volume	Mean volume Change in volume on OCT [1]	Macular diseases	Rapid, standardised and widespread; greater area of evaluation than central subfield thickness	Not highly correlated with VA; related to the timepoint assessed
Foveal avascular zone size	Change in area and/or perimeter on OCT-A [20]	DMO, DR, retinal vascular diseases	Rapid and precise	Related to the device used and image quality; absence of shared normal reference values
Vessel density/perfusion	Change in capillary perfusion/density on OCT-A [20]	DMO, DR, retinal vascular diseases	Precise	Related to the device used and image quality; absence of shared normal reference values

*AMD* age-related macular degeneration, *DMO* diabetic macular oedema, *DR* diabetic retinopathy, *OCT* optical coherence tomography, *OCT-A* optical coherence tomography angiography, *VA* visual acuity.

### Functional endpoints

Best corrected visual acuity (BCVA) is the most commonly used measure of visual function in clinical practice and the recommended primary endpoint in clinical trials for ocular conditions, recognised by the European Medicines Agency and the US Food and Drug Administration [1]. By quantifying the minimum visual angle of resolution, visual acuity (VA) provides a single measurement of a patient’s visual function [1, 17, 18]. The Snellen eye chart is a widely used method in clinical practice for the assessment of VA. However, due to its limited reliability and repeatability, Early Treatment Diabetic Retinopathy Study (ETDRS) charts are now required for registration trials. These charts were developed to quantify changes in vision due to panretinal photocoagulation in patients with DR [19]. They have a logarithmic progression of letter size and standardised chart lighting and seating distance from the chart [20, 21]. Continuous data (a score) are provided which are easy to analyse and can be used to make comparisons. It is determined by the patient reading the letter chart from a distance of 4 metres or more with an equal number of letters on each line and equal spacing of lines [21]. The change in BCVA is currently the most commonly used metric to assess visual function in eye disease. Improvement in BCVA is considered to be clinically meaningful when the mean visual angle doubles in resolution capacity. On a standard ETDRS chart, this change is equivalent to three lines (15 letters) [3]. A difference between groups in mean VA of 15 letters or more is also considered clinically significant [20, 21]. One of the main limitations of this functional metric is its value in assessing functional deficits in early disease and tracking small but important amounts of progression. Additionally, results are not necessarily parallel to disease progression as BCVA is only affected in specific clinical stages (e.g., when the disease involves the fovea) [17, 20]. BCVA also cannot detect changes over short trial periods, which may limit its use in clinical trials of high-prevalence diseases such as AMD and DMO [20].

Low-luminance VA, which is obtained by testing BCVA in dim light through specific filters, assesses central cone-mediated function under reduced luminance conditions. It has been proposed as a functional endpoint for AMD and inherited retinal diseases, as it is compromised early and may predict subsequent BCVA loss [1, 22]. Moreover, low-luminance VA, like BCVA, is strictly related to patient quality of life and disability [1, 23]. However, it primarily tests foveal function, thus providing select information [20].

Contrast sensitivity is the ability to detect boundaries or transitions between areas of relative darkness and relative lightness, and its impairment may precede BCVA loss in inherited retinal diseases, as well as in DR and DMO [1, 22]. It has shown to be strongly related to patient performance in daily activities; however, it still lacks standardisation [22, 24]. Moreover, it has been suggested to be hard to replicate the same exact conditions of execution and to have limited sensitivity to ischaemic changes [20].

Perimetry has been used in screening and monitoring to assess visual function in patients with retinal diseases [1]. The global indices of visual function (e.g., mean sensitivity and mean deviation) have already been included in clinical trials, and new parameters and methods of analysing the sensitivity data have been proposed with a more uniform and comprehensive evaluation of retinal sensitivity, such as Visual Field Modeling and Analysis [1]. However, perimetry requires adequate instruments and time, and may be influenced by the conditions of execution and the patient’s learning curve.

Microperimetry has the advantage of combining perimetry testing and fundus imaging for the study of macular sensitivity in correlation with the macular structure. It also allows for analysis of the fixation location and stability of a patient [1]. It has been applied to different retinal diseases; however, limitations include the cost of the device, the relative length of the examination, and the learning effect [18]. Finally, accurate visual field measurements require the ability to see and maintain fixation [3].

Dark adaptometry measures the length of time it takes for the retina to regain maximal sensitivity after it has been exposed to bright light. It provides a measure of visual cycle as a slowing of the photoreceptors’ recovery, which is suggested to be linked to AMD onset and progression [20, 23]. However, dark adaptometry requires a significant amount of time to implement and has mainly been proposed as a complementary metric [23, 25].

Patient-reported outcome endpoints (e.g., EuroQoL 5 Dimensions, NEI-VFQ-25 [25-item National Eye Institute Visual Function Questionnaire]) have gained increasing relevance both in clinical practice and clinical trials, as they are used to support claims in medical product labelling approvals and have been suggested to be more meaningful to the patient compared to conventional endpoints [26]. Questionnaire tools focused on a patient’s subjective experience may be applied to several common eye conditions, including age-related cataracts, AMD, DR and glaucoma. However, it is still difficult to delineate how much of an improvement based on survey responses would be clinically meaningful. In addition, the repeatable assessment of patient quality of life in a clinical trial is still challenging [1].

The significance of and consistency in evidence for all the aforementioned functional parameters is growing, and some have already been included in both clinical trials and clinical practice [1, 22]. However, most of the parameters, besides BCVA, are still used as secondary or composite endpoints, which provide information on the early stages of a disease, necessitate specific statistical requirements, and are often associated with a learning curve for patients requiring repeated measurements [2]. Other functional parameters are needed for the late stages of chorioretinal diseases when visual function is severely reduced. Methods such as full-field stimulus threshold, pupillography, electroretinography and reading speed have been studied and even included in some trials, but in limited areas of application [1, 27]. Finally, an important shortcoming of all these biomarkers, including BCVA, is the lack of information on disease course, which has a primary role in the prognosis of the most prevalent retinal diseases involving the posterior pole, such as DMO and AMD – the main focus of this review [4]. TIR might be applied on every numerical outcome with multiple measurements, thus, to all clinical mentioned endpoints.

### Morphological metrics

It has been suggested that clinical trials may benefit from a combination of functional and morphological endpoints, such as the onset of proliferative DR or changes in macular centre thickness, as measured by OCT [21]. Morphological markers may have important pathophysiological significance, showing how the different structures are modified during the disease and treatment. The correlation between imaging metrics and biochemical biomarkers may give significant insight into the meaning of different morphological markers [28–30].

Several morphological metrics have been proposed for monitoring ocular diseases. Diagnostic imaging modalities such as fundus autofluorescence and OCT are recognised clinical techniques providing standardised endpoints in clinical trials [1]. The strong advantages, particularly of OCT, and more recently OCT-A, in terms of their non-invasiveness, standardisation and rapidity of execution have made them particularly promising for the development of clinical biomarkers in chorioretinal diseases [31, 32].

OCT has shown to be able to detect early features of atrophy (incomplete retinal pigment epithelial and outer retinal atrophy) and changes in the status of the ellipsoid zone and external limiting membrane. Ellipsoid zone defects are believed to be the product of photoreceptor loss, and are strongly correlated to VA [33]. Changes in the ellipsoid zone and external limiting membrane status have been proposed and recently used as an endpoint in clinical trials [20, 34, 35]. Currently, however, the change in area of loss of blue light fundus autofluorescence is still recognised as a primary outcome in geographic atrophy treatment trials by the US Food and Drug Administration and European Medicines Agency, rather than OCT changes.

The most frequently used OCT biomarker, particularly in exudative retinal diseases but not exclusively, is central subfield thickness (CST), defined as the mean thickness in the central area with 1 mm diameter centred onto the fovea [36]. CST is widely used in clinical practice and as a standardised secondary endpoint in most clinical trials [37, 38]. However, the correlation between CST and BCVA has been shown to be weak to moderate in different diseases [39–41]. The total macular volume has been suggested as a possible more useful parameter to better describe the extent and severity of the oedema. Use of both CST and the volume may provide a more complete picture in most cases [1]. Moreover, the presence (and absence) of intraretinal and subretinal fluid has been gaining in relevance and the fluid-free interval has been proposed as a possible endpoint [21]. At present, OCT measures of macular oedema are still mainly used as surrogate endpoints in clinical trials [21]. Furthermore, not all aspects of retinal diseases may be fully detected by OCT. For example, the Diabetic Retinopathy Severity Scale score has been introduced as a primary endpoint in studies evaluating therapies for non-proliferative DR [1].

Besides those previously mentioned, several other imaging biomarkers (in particular, OCT) have been proposed in the literature, including the disorganisation of retinal inner layers [33]. However, disorganisation of retinal inner layers still lacks standardisation in terms of its detection and measurement, and also lacks a recognised pathophysiological meaning [42, 43].

OCT-A can provide quantitative and repeatable metrics shown to be related to visual function [20]. The use of OCT-A-based parameters of vascularisation (e.g., foveal avascular zone, vascular density, vascular perfusion) may predict the development of macular oedema and those of macular choroidal neovascular lesions may help to monitor them [1, 20]. Larger prospective studies are necessary to best define their role [1, 20]. Compared with OCT, OCT-A seems to be more prone to artefacts and to higher variability in the definition of proposed metrics among the studies and devices [20]. Further limitations of OCT and OCT-A include the variability of measurements from the multiple instruments available and the quality and focalisation of images [20]. Finally, all the aforementioned metrics describe features detected at a precise moment in time and not their evolution over time. The application of the TIR concept to morphological parameters may add extra value.

## Application of the TIR concept in retinal diseases

The advent of intravitreal therapies has dramatically changed the treatment of major retinal diseases, namely DR, DMO and AMD. It has opened a wide range of new therapeutic perspectives requiring both formal validation and clear clinical guidelines for the correct indication and monitoring [1]. As a result, the need for adequate endpoints has emerged.

Monitoring the patient’s response to treatment is a key aspect of the management of retinal diseases, since their pathophysiology and phenotypes are complex and the responses after treatment may vary significantly between patients and according to the disease, its characteristics (e.g., lesion type in AMD), severity, activity and concomitant factors (e.g., glycaemic control in DMO) [44, 45]. In neovascular AMD, the presence of retinal thickness fluctuations with re-accumulation of fluid at some visits significantly reduced the final VA compared to stable patients, even after correction for age, lesion size and type, foveal thickness, and intraretinal fluid at baseline [44]. The same effect of retinal thickness fluctuations on VA has been found in DMO and retinal vein occlusion despite intravitreal treatment [45]. These results were obtained in a clinical trial setting, and thus we may assume that the effect could be even greater in real-world clinical practice where higher variability in the timing of treatments and follow-up may increase the fluctuation effect.

The US Food and Drug Administration recommends evaluating statistically and clinically relevant differences in visual function at more than one timepoint [21]. However, even this type of metric does not provide a unique measure of fluctuations in VA during the examined period.

At present, the most widely accepted and meaningful clinical marker for ocular disease is BCVA [1]. Mean VA change has the advantage of considering both improvement and worsening, but still only provides information on a single timepoint and not the whole disease course. The area under the curve has been used to give a more complete measure of VA (but also of other endpoints such as CST) over a period of time, for example in patients with DMO treated with intravitreal anti-vascular endothelial growth factor (VEGF) [5]. However, this type of analysis only provides an average measure and does not assess the time spent within the certain value of a metric presumed to be a ‘safe range’ [4]. While it has been used to assess performance among drugs, it has not provided an individual’s performance on a certain medicine. Moreover, the area under the curve can sometimes be difficult to interpret, providing data that are not intuitive for everyone [4, 5].

Recently, the concept of TIR has been applied in ophthalmology (particularly in DMO), allowing the consideration of fluctuations in visual function that are characteristic of the exudative nature of the oedema secondary to diabetes [4]. The current evidence is based on post-hoc analysis and, to date, no prospective clinical trials have formally evaluated TIR as a primary or secondary endpoint in retinal diseases [4, 46, 47]. Calculation of TIR requires frequent, standardised assessments, which may be challenging in real-world clinical settings where visit frequency and image quality vary. Future studies are needed to confirm the potential of TIR to allow clinicians to determine the time (e.g., in weeks) or proportion of time (as a percentage) in which a patient or group of patients maintains a certain value (range) of a metric [7]. They are also necessary to confirm the reproducible statistical consistency of TIR analysis with already recognised endpoints, as well as its ability to implement the information obtained from other endpoints. These points are crucial to validate TIR as an endpoint recognised by the regulatory agencies.

Designing a trial using TIR would require careful sample size calculation, which depends on the expected distribution of the TIR variable, its variance and the clinically meaningful difference to detect. Compared to single timepoint measures, TIR introduces complexity due to its longitudinal nature. However, repeated measurements are already required in clinical trials for major retinal diseases to assess the course of disease and response to therapies. No additional test or evaluation needs to be added in the patient’s visit, and TIR does not add possible reproducibility bias and inter-operator variability to standard endpoints, since the data analysed are not modified. Strategies for handling missing data should be preplanned, for example with careful sensitivity analysis [48].

The functional endpoints and morphological metrics reviewed, including low-luminance VA, contrast sensitivity, perimetry and microperimetry indices, can all be evaluated using the TIR concept; for each, an adequate threshold to consider over a certain period of time is set. Application of the TIR concept to retinal diseases could help overcome the current lack of information on disease course, but it would not address the specific existing limitations of each metric, such as the focus of low-luminance VA on foveal function or the lack of standardisation of contrast sensitivity tests [24, 27].

Therefore, at present, due to its characteristics, a VA threshold appears to be the best metric to apply to the TIR concept from the perspective of a clinical endpoint. A score of 69 ETDRS letters or 20/40 has been considered a cut-off between good and worse VA in clinical trials [49, 50]. Moreover, it is the most frequently recognised target required for holding a driving licence. Therefore, it appears to be an adequate threshold to delineate the area of autonomy of patients [51]. In a post-hoc analysis of the Protocol T trial, different BCVA thresholds were proposed for the application of TIR during intravitreal treatment for DMO, from ≥30 to <90 letters, and the results were consistent with those obtained using the 69-letter threshold [4]. In the same study, the number of BCVA assessments in the first year of treatment was set at greater than seven (and greater than four for the second year), corresponding to the dosing regimens of anti-VEGF agents and excluding eyes with fewer measurements [4]. The seven-point profile (consisting of seven repeated measures) has already been used for TIR of glucose levels in diabetic patients [9]. Yet, TIR could also include a variety of measurement profiles, according to the specific clinical setting studied: short-term fluctuations within days, longer-term fluctuations over months or time until vision goes below a certain level [4].

The BCVA TIR metric used in the post-hoc analysis of Protocol T evaluated the differences between various anti-VEGF drugs. The results were in line with the letter gains in BCVA over the course of the study, which was the prespecified primary outcome of the trial [4]. In particular, TIR (at a BCVA letter score of ≥69) in the first 52 weeks was 41, 38 and 37 weeks for aflibercept, ranibizumab and bevacizumab treatment, respectively [4], which is in line with the BCVA increases of 18.9 ± 11.5, 11.8 ± 12.0 and 14.2 ± 10.6 ETDRS letters reported for the same treatment groups at year 1 [50]. The analysis by Kozak et al. confirms the consistency of the TIR metric, provides a more complete measure of the VA data obtained during treatment and better reflects the lived experience of patients with DMO [4]. The expression of the gain or loss obtained in terms of percentage of time with ‘good’ vision may be an intuitive concept to better communicate with patients and more easily explain why a specific timing of treatments (e.g., injections) is preferred [7].

Moreover, by linking TIR with patient-reported outcomes, such as the NEI-VFQ-25 or other vision-related quality-of-life instruments, we can gain a more holistic understanding of how maintaining 'in-range' status translates into functional benefits perceived by patients. This integration may allow, for example, the validation of clinically meaningful TIR thresholds (e.g., BCVA ≥ 69 ETDRS letters) based on their correlation with improved daily functioning and quality of life and the development of composite endpoints combining objective TIR metrics with subjective patient experience, which could support regulatory and clinical decision-making.

The application of TIR in diabetes monitoring has already shown to improve the clinical management of patients affected by type 1 diabetes. The results from the GOLD and SILVER studies showed that inter-individual variations exist between TIR and HbA1c and the detection of high HbA1c without considering that glycaemia fluctuations may lead, for example, to an increased risk of hypoglycaemia [52]. TIR has also been recently used as a primary endpoint in the SWITCH PRO study and in the InRange comparison trial for the evaluation of the efficacy of two insulin basal analogues [53, 54].

In DR, the analysis of data from the Diabetic Retinopathy Clinical Research Retina Network’s Protocol S using bootstrap simulation showed that TIR was better correlated with final outcomes rather than changes from baseline, highlighting its role in assessing long-term disease stability rather than short-term fluctuations [47]. This seems to confirm TIR as a complementary outcome measure alongside traditional metrics. However, this remains a hypothesis-generating framework until more robust data are available.

TIR has been shown to be applicable to DMO and is also expected to be applicable to other retinal diseases such as AMD [4]. The potential for the application of TIR to a variety of retinal and ocular diseases could result in it becoming a widespread metric and thus being well understood by clinicians and researchers.

Implementing TIR metrics in routine clinical practice in ophthalmology first needs validation but can potentially improve the understanding of disease progression or response to therapy, thus allowing a personalised and more tailored healthcare pathway. It may be particularly useful to better understand the course of the disease in patients with poor or no response to therapy. TIR calculation does not require any additional evaluation at the scheduled visits: the measurements are analysed over a period of time, with last observation carried forward imputation for missing visits. The percentage of time spent below or above a threshold may be implemented in the treatment decision-making process. This is particularly relevant in the present day and will be even more relevant in the future with the advent of a larger number of therapeutic options. In fact, the post-hoc analysis of randomised clinical trials using TIR may provide a more specific measure of the possible differences among drugs, allowing for a more targeted treatment strategy in clinical practice. Moreover, the TIR obtained, even in real-world practice, may allow us to better understand the response to a specific treatment and eventually the necessity of switching [48].

Recent significant advancements in retinal imaging diagnostic technologies have led to a growing relevance of morphological biomarkers in the management and better comprehension of the pathophysiology of retinal diseases. Hence, it would be logical to also apply the concept of TIR to the main recognised and standardised morphological biomarkers, such as CST and macular volume or fluid volume. These metrics may provide relevant information on the structural changes over time and their correlation with the course of VA during the disease [55].

Most recently, TIR has been applied to analyse the results of randomised clinical trials of patients treated with dexamethasone implant using central retinal thickness (CRT) thresholds of <300, <353, <446 and <551 μm. Steroid treatment was associated with significantly longer TIR than sham across all thresholds. TIR difference between groups was greater at lower CRT thresholds, with the maximal difference at the normal CRT cut-off of 300 μm with a median TIR of 18.5 weeks [46].

In this study, CRT was derived from OCT scans conducted at 3-month intervals, thus including four measurements. The results were consistent with standard metrics (e.g., BCVA and CRT change), showing the possible use of TIR even with fewer than the standard seven records [46].

Numerous measurements may further improve the results obtained, making TIR more informative. However, the collection of measurements may be limited by the current necessity of scheduling monitoring visits, which would increase the burden for the patients and the healthcare system [48]. The increased use of drugs that allow shorter loading dose and the possibility to progressively extend the intervals may see a reduction in the frequency of measurements, even in the first year, thus a more distributed number of assessments. Moreover, even with extended treatment intervals, regular, fixed visits will still be required to optimally monitor several treatable conditions and to assess treatments under development, both in clinical trials and clinical practice [48]. Therefore, TIR might be particularly informative when the use of home monitoring has become a concrete possibility for patients and when the telemedicine approach is more integrated in our clinical practice [56]. These methods would allow a significant number of measurements to be taken daily or multiple times per day, possibly leading to increased sensitivity of the metric.

Furthermore, while the application of TIR metrics may require more frequent assessments—potentially increasing upfront costs—these are offset by the potential long-term economic benefits. Improved disease monitoring through TIR may enable earlier detection of treatment failure, better individualisation of therapy and reduced risk of vision loss, which is associated with substantial societal and healthcare costs. Moreover, emerging home-monitoring technologies may offer cost-effective solutions that reduce the burden on clinical resources [48].

One of the main limitations of morphological markers is that they are disease- and imaging-related; in fact, different endpoints are usually reported for different diseases [1]. Therefore, their use seems to depend on the specific morphological features set as the primary outcome of a trial or of primary clinical interest in a patient. At present, the US Food and Drug Administration recommends using visual function as a primary endpoint in clinical trials assessing treatments for ocular diseases; therefore, morphological endpoints still need to be considered in combination with functional endpoints [21].

### Other applications in ophthalmology

While the focus of this review is retinal diseases, particularly exudative diseases involving the posterior pole, the concept of TIR may be easily applied to other eye conditions. The necessity of repeated measurements of intraocular pressure, even within the same day, is established in glaucoma [57]. TIR could provide an individualised and informative measure of the course of intraocular pressure over 12–24 h, providing data that might easily be correlated to the morphological and functional outcomes of the disease. Moreover, retinal nerve fibre layer changes (as measured by OCT) may predict future visual field losses. In particular, the initial rapid retinal nerve fibre layer thinning during early follow-up of patients with glaucoma has been shown to be strongly predictive of large visual field loss [1]. The possible quantification of the persistence, or not, of imaging parameters within a range could provide a new endpoint in glaucoma too. Similarly, in uveitic conditions, the time spent with inflammation control (clinical TIR) could be plotted for individual patients and/or compared between various treatments.

### Limitations

This framework is currently limited by its reliance on post-hoc analyses, lack of standardisation in TIR thresholds and need for frequent data collection. Prospective validation studies are necessary to assess feasibility, patient relevance and correlation with long-term outcomes.

At present, the lack of prospective studies specifically designed to assess TIR as a primary endpoint, even in retinal diseases, limits the full understanding of its potential.

The identification of a more complete metric as an endpoint in clinical trials remains a priority in the process of standardising endpoint measures aimed at better understanding the course of ocular diseases and their response to treatment, as well as their clinical management.

## Vision Academy recommendations

- When evaluating disease course and treatment response, the limitations of reporting a one-time endpoint should be recognised.
- Future analyses should consider, among other metrics, the BCVA TIR (i.e., the percentage of time the patient had a BCVA above a certain threshold).
- TIR may be applied to morphological parameters as additional metrics to the functional ones.

These recommendations were formulated by the authors of this article and submitted to the entire Vision Academy membership for endorsement; 57 responses were received. Overall, the recommendations were endorsed by 92% of respondents (a response of ‘agree’ or ‘strongly agree’), with the level of endorsement for each individual recommendation ranging from 88% to 96%. The mean (range) rate of non-endorsement was 1% (0–2%) for a response of either ‘disagree’ or ‘strongly disagree’, and 6% (2–11%) for a response of ‘neither agree nor disagree’.

## Conclusions

Choosing proper efficacy endpoints plays a key role in the overall design of a clinical trial and the future of investigational treatments. Although a vision endpoint is the most important determinant of drug efficacy, novel endpoints, including morphological ones, are meaningful in the design of ongoing and future clinical trials assessing treatments for retinal diseases.

BCVA TIR may provide crucial information on the primary outcome of any clinical trial in ophthalmology and overall visual function, not only at the end of a process/treatment but also as visual fluctuations occur during the course of a disease. The TIR concept may also be useful in clinical practice as a measure of the course of disease in a single patient. It may additionally be applied to other endpoints, such as morphological and clinical endpoints for ocular diseases.

TIR is proposed not as a replacement for existing endpoints such as BCVA, but as a complementary measure that adds temporal insight into disease control and treatment response.
